# Roles of different types of oxalate surface complexes in dissolution process of ferrihydrite aggregates

**DOI:** 10.1038/s41598-018-20401-5

**Published:** 2018-02-01

**Authors:** Fengyi Li, Luuk Koopal, Wenfeng Tan

**Affiliations:** 1 Key Laboratory of Arable Land Conservation (Middle and Lower Reaches of Yangtze River), Ministry of Agriculture, College of Resources and Environment, Huazhong Agricultural University, Wuhan, 430070 People’s Republic of China; 2 Physical Chemistry and Soft Matter, Wageningen University and Research, Stippeneng 4 (Helix), 6708 WE Wageningen, The Netherlands

## Abstract

The dissolution of ferrihydrite induced by low molar mass (LMM) organics is an important process that provides bioavailable iron for organisms. Here, ATR-FTIR analysis was combined with characterization of ferrihydrite nanoparticles and kinetic modeling to investigate the roles of different oxalate surface complex species in the dissolution of ferrihydrite aggregates. ATR-FTIR results revealed that at least four different species were present at or near the ferrihydrite surface in the process of ferrihydrite aggregate dissolution. At a relatively low addition of oxalate (oxalate/Fe < 0.1), oxalate was dominantly present as binuclear bidentate surface complexes and aqueous species. The binuclear bidentate complexes mainly caused electrostatic repulsion between particles, resulting in the disaggregation of large ferrihydrite aggregates into colloidal particles with hydrodynamic diameters of 116–174 nm. Kinetic modeling showed that these colloidal particles were stable at the oxalate/Fe ratio of 0.1. With increasing addition of oxalate (oxalate/Fe ≥ 0.1), mononuclear bidentate oxalate complexes and hydrogen-bonded surface complex replaced the binuclear bidentate complexes and aqueous species. The aggregates or larger colloidal particles were further disaggregated into smaller colloidal particles with hydrodynamic diameters of 35–64 nm. Additionally, the mononuclear bidentate oxalate complexes promoted the dissolution of ferrihydrite colloids into dissolved Fe.

## Introduction

The dissolution of iron (hydr)oxides is an important process providing bioavailable iron for plants and microorganisms and affecting the fate of contaminants in the environment^1–4^. As the main components in the exudates of plant roots or microorganisms, low molar mass (LMM) organic anions are commonly found in natural environments^5,6^. They are actively involved in the dissolution processes of iron (hydr)oxides^7–12^ and markedly increase the dissolution extent and Fe release rate^13–18^.

In the studies of the kinetics and extent of iron (hydr)oxide dissolution, the processes are typically defined as non-reductive or reductive. Non-reductive dissolution processes are enhanced by protons or organic ligands^19^. Proton-enhanced dissolution is important in acidic environments, because high concentrations of protons can weaken the metal-oxygen bonds in the crystal lattice, leaving metal cations susceptible to liberation from the surface^20^. In near nexutral environments, (hydr)oxide dissolution requires organic ligands because the formation of metal-organic surface complexes polarizes the metal-oxygen bonds and lowers the energy barrier for dissolution^21^. Detailed investigations showed that the effects of organic ligands on the kinetics and extent of dissolution are closely related to their surface coordination modes of the LMM organic ions^22–30^. Taking carboxylates as an example, they can form inner-sphere complexes with metal oxides of seven-, six-, and five-membered chelate ring structures^29,30^. A number of evidences show that the surface complexes with five-membered chelate ring structures have better dissolution-enhancing capacity than those with six- and seven-chelate ring structures^22,29,31^. For this phenomenon, the generally accepted explanation is that the stabilizing effects against dissolution imparted by the complexes with seven-ring structures can be largely attributed to the low probability of simultaneous detachment of two metal cations from mineral surfaces^32^. Additionally, some attention has been paid to the influence of outer-sphere complexes of LMM organics on metal (hydr)oxide dissolution. However, no consensus has been reached to date as the conclusions are contradictory^22,29^.

Reductive dissolution of iron (hydr)oxides, which is important in some natural systems, has also been discussed in detail^33^. Reductive dissolution is typically associated with photochemical or biological reactions. As for photochemical reactions, the two key steps are photoreduction of Fe(III) at the (hydr)oxide surface and subsequent release of more soluble Fe(II) into the solution^16^. In the presence of organic ligands, Fe(II) is generated with higher efficiency via a ligand-to-metal charge-transfer reaction induced by light within the metal-organic surface complexes, followed by the detachment of Fe(II) from (hydr)oxide surface with the help of certain ligands specific for binding Fe(III), such as desferrioxamine B, DFOB^13,34,35^. A previous investigation has provided spectroscopic evidences for the photoreductive dissolution of lepidocrocite in the presence of citrate, suggesting that such dissolution process is to some extent controlled by the coordination modes of metal-organic surface complexes, because different surface coordination modes of citrate show different photoreactivity^16^.

Several variables, which link macro-scale dissolution with molecular-scale complexation, have been well documented, including solution pH, types of organic ligands, and particle size of iron oxides^22–30^. Another important factor that remains to be fully explored is the influence of the inherent mineralogical properties of iron (hydr)oxides on the dissolution processes induced by LMM organics. Several recent studies have demonstrated that the size, morphology, and aggregation state of iron (hydr)oxide nanoparticles impact their dissolution degree and kinetics^20,36–39^. For instance, the aggregation state of 7-nm hematite particles significantly affected the initial and steady state dissolution rate in the presence of ascorbic acid^37^.

To date, large efforts have been made to understand the effect of the particle aggregation state on the dissolution rates of iron oxides, but relatively few studies focus on the roles of surface complexes of LMM organic acids in the dissolution process of aggregates. Thus, this work aims to investigate the relationships of different surface complex species of LMM organics with the dissolution behavior of iron (hydr)oxide aggregates. For this purpose, the ferrihydrite-oxalate system was selected as the research subject. Ferrihydrite is recognized as one of the most reactive iron oxides and plays an important role in controlling the transport of environmental contaminants like arsenic and lead^40^. The average size of individual ferrihydrite particles is about 2–7 nm, but they usually exist in the form of aggregated structures in the environment^41,42^. Oxalate is the smallest organic dicarboxylate ligand among LMM organic compounds and is also one of the most abundant LMM organic acids in nature^5^. Its concentration in natural systems ranges from 2.5 × 10^−5^ to 4.0 × 10^−3^ M^2^. Fourier transform infrared spectroscopy (ATR-FTIR) was used to obtain detailed structural information of the oxalate species adsorbed on ferrihydrite. Similar experiments have been performed to quantify the influence of surface speciation of oxalate on boehmite dissolution^28,29^. Results from these ATR-FTIR studies were then combined with the results from various characterization methods to provide molecular-level insights into the impacts of different oxalate surface complex species on the dissolution of ferrihydrite aggregates. These different methods of characterization include X-ray diffraction (XRD), transmission electron microscopy (TEM), dynamic light scattering (DLS), and mathematical modeling of the dissolution kinetics.

## Experimental Methods

### Chemicals

All chemicals were of analytical grade and were obtained from Sinopharm Chemical Reagent Ltd. Corp., and were used without additional purification. Solutions and suspensions were prepared using 18 MΩ-cm Millipore water.

### Ferrihydrite preparation and characterization

A 1 mol/L NaOH solution was added at a rate of 1 ml min^−1^ to 0.1 mol/L Fe(NO_3_)_3_ solution under constant stirring using a computer-controlled Titrando 836 titration system, until the pH reached 7.0 ± 0.2. The pH of the suspensions was stabilized for 2 h and readjusted to pH 7.0 ± 0.2, if necessary. The precipitate was then washed several times to remove excess NaNO_3_, and subsequently freeze-dried and stored in a desiccator. In this study, all experiments were finished within 10 days to minimize the possibility of mineral phase transformation.

The samples were characterized X-ray diffraction (XRD), transmission electron microscopy (TEM), Brunauer-Emmett-Teller (BET) surface area analysis, and dynamic light scattering (DLS). The diffraction pattern was collected using a Bruker D8 diffractometer using Cu kα radiation (λ = 1.5406 Å) at 40 kV and 40 mA. The diffractograms were recorded from 5 to 80° with 0.01° 2θ steps and 1° per 1 min. The XRD data were further analyzed by the Rietveld refinement method; the fitting model was obtained from Michel (ICSD 158475). TEM images of the samples were collected using a JEOL JEM 2100 operating at a voltage of 200 kV. About 5 mg of sample was suspended in 10 ml ultrapure water and sonicated for 20 min. Subsequently a drop of the suspension was placed on lacey carbon-coated cooper grid and dried under ambient conditions.

Specific surface areas were determined by N_2_ gas adsorption using a Quantachrome Nova 4200e surface area analyzer and the multi-point BET method. Samples were outgassed for 12 h at relatively low temperature (60 °C) to prevent the transformation from ferrihydrite to goethite or hematite. Specific surface areas were reported as the average of triplicate measurements.

### Preparation and speciation calculation of aqueous oxalate-Fe(III) complexes

A 50 mL 1 mmol/L oxalate solution (H_2_C_2_O_4_·2H_2_O) was mixed with 50 mL 1 or 2 mmol/L ferric nitrate solution (Fe(NO_3_)_3_·9H_2_O). Then, 0.1 mol/L NaOH was added to the mixture until the pH reached 2.0 ± 0.2. The speciation was calculated using Visual MINTEQ. 3.1 software.

### Dissolution experiments

Ferrihydrite dissolution batch experiments were carried out in triplicates with different oxalate concentrations. First the dissolution at pH 4.5 was compared with that at pH 7, together with the effect of ultrasonic dispergation prior to the start of the dissolution. The experiments at pH 7 were carried-out in the same way as those at pH 4.5, except for the pH and only the dissolved Fe concentration was followed over a period of 100 h. Before dissolution, ferrihydrite was acidified to pH 4.5, and then purged overnight with N_2_ to remove CO_2_. 25 mL of ferrihydrite stock suspension (120 mmol Fe/L) and 25 mL pH 4.5 oxalate solution with 10 mmol/L NaCl were added to a series of polypropylene centrifuge tubes (100 mL), and four different oxalate solutions were used covering a concentration range of 1.2 to 60 mmol/L, resulting in the oxalate/Fe molar ratios of 0.01, 0.1, 0.2 and 0.5, respectively. The tubes were wrapped in Al-foil to avoid photo-induced reduction, and were rotated in an incubator shaker at room temperature. For each oxalate/Fe ratio the dissolution reaction was investigated after 2, 4, 8, 12, 24, 48, 60, 72 h, respectively. The samples were divided into three aliquots: two were individually passed through filters with pore sizes of 0.45 and 0.22 μm, respectively, and in each filtrate the concentrations of dissolved Fe and oxalate were measured together with the average hydrodynamic diameters of the particles present in the filtrates. The hydrodynamic diameters were obtained with Dynamic light scattering (DLS). The filtration with the 0.45 μm filter was carried out to remove the very large aggregates that strongly affect the average hydrodynamic diameters. Compared with the 0.45 μm filtrate, the 0.22 μm filtrate was clear, suggesting that most suspended particles and aggregates were removed from the suspension. Also in previous studies, 0.22 μm nylon filters were used to remove suspended ferrihydrite aggregates and particles from acidic aqueous solution^37,43,44^. The third aliquot was centrifuged at 10000 rpm for 60 min to separate and concentrate the pastes for ATR-FTIR analysis (see the next section).

Total dissolved Fe(III) was measured by an UV–vis spectrophotometer (UV-2450, Japan) at the wavelength of 510 nm, using the 1, 10-phenanthroline method^45^. Oxalate in the filtrates was measured by Leco CHNS-932 elemental analyzer after acidification of the samples with 5% (v/v) 30% HCl.

Dynamic light scattering (DLS) measurements were carried out with a Malvern Zeta-sizer (ZEN 3600 analyzer) to obtain the average size of the ferrihydrite aggregates in the filtrates (0.45 μm filter and 0.22 μm filter) of the samples after 72 h of dissolution. The measurements were carried out in the low volume quartz cuvette. Sample measurements, composed of 10 runs, were conducted with a collection time of 10 s per run. The average particle size obtained from three of such measurements was reported. The Fe concentration in samples for DLS measurement ranged from 0.04–0.25 g/L.

### ATR-FTIR spectroscopy

ATR-FTIR spectra were collected with a Bruker Vertex 70 FTIR spectrometer equipped with a deuterated triglycine sulfate (DTGS) detector. A single-reflection diamond ATR accessory (Pike Technologies, Inc., Madison, WI, USA) was used to acquire the spectra of wet pastes. The samples were uniformly applied directly to diamond ATR crystal, and the sample-holding region was covered with a glass lid to prevent water evaporation during data collection. For each spectral measurement, 512 scans were recorded in the wavenumber range from 1000 to 2000 cm^−1^ at a resolution of 4 cm^−1^. The raw spectra of the wet paste were dominated by the strong contributions from water. To isolate the spectra of the solid phase, the paste spectra were subtracted from the spectra of supernatants to remove the strong contributions from the water bands.

Two-dimensional correlation spectroscopy was employed to investigate the transformation between different surface complex species. Prior to the 2D analysis, the FTIR spectra in the wavenumber range of 1800 to 1200 cm^−1^ from 2 to 72 h were baseline-corrected and smoothed. The average spectrum was used as a reference. Practical computation of the 2D FTIR correlation analysis was performed using the 2D Shige software^46^.

The 2D correlation procedure generates two types of plots: synchronous, Φ (Ѵ_1_, Ѵ_2_) and asynchronous, Ψ (Ѵ_1_, Ѵ_2_), where Ѵ_1_ and Ѵ_2_ are band frequencies. In the synchronous plot, the auto peak (Ѵ_1_ = Ѵ_2_) is responsible for the change of peak intensity due to the external perturbation, while the cross peak (Ѵ_1_≠Ѵ_2_) gives the correlation among the auto peaks. For the cross peak, the same sign of two bands at Ѵ_1_ and Ѵ_2_ indicates simultaneous change of peak intensity, and the opposite sign indicates the increase of the intensity of one peak at the expense of the other. In the asynchronous plot, the cross peak arises when Ѵ_1_ and Ѵ_2_ change independently of each other. The sequential order of intensity change between two bands at Ѵ_1_ and Ѵ_2_ obeys Noda’s rule. In brief, if Φ and Ψ have the same sign, the intensity change at Ѵ_1_ occurs prior to that at Ѵ_2,_ while if Φ and Ψ have the opposite sign, the order is reverse^47^.

### Modeling of the dissolution kinetics

The mathematical modeling of the ferrihydrite dissolution kinetics induced by oxalate was based on the modified Noyes-Whitney equation, as described in the work of Braunschweig *et al*.^38^. Briefly, the dissolution process of the ferrihydrite is divided in three steps: (1) dissolution of the primary ferrihydrite particles with extremely small sizes into dissolved Fe; (2) partial disaggregation of large ferrihydrite aggregates into smaller colloidal particles; (3) dissolution of the colloidal particles into dissolved Fe. Thus, the above process can be mathematically described as:

1 $$\frac{dFh}{dt}=-{k}_{1}\times Fh-{k}_{2}\times {(Fh)}^{2},$$

where [Fh] represents ferrihydrite aggregates, which is the initial Fe concentration added in the batch experiments. k_1_ and k_2_ [h^−1^] represent the growth rate constant of colloids and the dissolution rate constant of ferrihydrite aggregates, respectively. Eq. (1) suggests the direct ‘dissolution’ of ferrihydrite aggregates into dissolved Fe and colloids.

2 $$\frac{dF{e}_{col}}{dt}={k}_{1}\times Fh-{k}_{3}\times F{e}_{col}\times (O{x}_{max}-Fh),$$

where [Fecol] represents total colloidal Fe. k_3_ is the dissolution rate constant of colloids. Eq. (2) suggests the further dissolution of colloidal fraction into dissolved Fe. The process depends on the difference between the initial (maximum) oxalate concentration Ox_max_ and the concentration of ferrihydrite aggregates. The values for the initial oxalate concentration [Ox_max_] were based on the oxalate concentrations added in the batch experiments.

The total dissolved Fe-oxalate can be described as:

3 $$\frac{dF{e}_{diss}}{dt}={k}_{2}\times {(Fh)}^{2}+{k}_{3}\times F{e}_{col}\times (O{x}_{max}-Fh),$$

where the first contribution is the dissolution of the primary particles, the second the dissolution of the colloidal fraction that was produced by disaggregation. The system of differential equations (1)–(3) can be solved numerically for the three k values (k_1_, k_2_, k_3_) by minimizing the weighted sum of squared deviations (SQR) between the measured and modeled dissolution values using a nonlinear least-squares regression.

## Results

### Characteristics of the ferrihydrite sample before dissolution

The powder XRD pattern in Figure S1 showed that the product was “2-line” ferrihydrite with its characteristic reflections at d = 0.15 nm and 0.25 nm. The crystallite size obtained from the Rietveld refinement result was 2.6 nm. TEM image in Figure S2a showed that the sample appeared as particle aggregates with a smooth surface texture. According to the HRTEM image in Figure S2b, the sizes of the primary ferrihydrite nanoparticles were about 2.5–5 nm, and the particles could be considered to have grain-to-grain contact in the aggregates.

The BET specific surface area of ferrihydrite aggregates was 300 ± 30 m^2^/g. The geometric specific surface area, SSA_GEO_, was estimated using Eq. (4) with the assumption that the ferrihydrite particles were spherical:

4 $${{\rm{SSA}}}_{{\rm{GEO}}}=\frac{6}{{\rm{\rho }}\cdot d},$$

where ρ is the density of ferrihydrite and d is diameter. In this study, ρ was obtained from the work of Hiemstra and Zhao^48^, and *d* (=2.6 nm) was obtained from Rietveld refinement results. The calculated SSA_GEO_ of the ferrihydrite nanoparticles was 607 m^2^/g. The SSA_BET_ of ferrihydrite was only about half of SSA_GEO_, suggesting a dense aggregation of primary particles in freeze-dried ferrihydrite. Stegemeier *et al*.^49,50^ pointed out that the factors that enhance iron oxyhydroxides nanoparticle aggregation generally follow the order of ionic strength ≈ pH < aging < freezing < drying. Accordingly, the interstitial water and pore space between aggregated particles are decreased by these factors and result in greater compaction of the aggregates and smaller specific surface areas in the same order. These conclusions supported our results of the specific surface area and the observations from the HRTEM images.

### Kinetics, characteristics and mathematics modeling of the dissolution

Previous research regarding oxalate-promoted dissolution of ferrihydrite has shown that proton-promoted dissolution at pH 4.0 was negligible^15^. In order to check the effect of pH on the dissolution of the present ferrihydrite aggregates, two sets of experiments were carried-out, one at pH 4.5, and the other at pH 7. In addition to the effect of pH, the effect of aggregation state was also determined in these experiments. For both experiments, one set of ferrihydrite suspensions was dispersed for 30 min by ultrasonic dispergation prior to the dissolution at the given pH and oxalate/Fe ratios of 0, 0.01, 0.1, 0.2 and 0.5; the other suspensions were directly subjected to dissolution. The corresponding results are shown in Figure S3.

For the experiment at pH 4.5, proton-promoted ferrihydrite dissolution was not observed in the absence of dispergation and oxalate at t < 12 h, and the total dissolved Fe was ~1 µmmol within 100 h. When the sample was dispersed ultrasonically, proton-promoted dissolution was observed in the absence of oxalate at <12 h, and the total dissolved Fe was ~1.1 µmmol at 12 h and ~1.9 µmmol at 100 h. In the presence of oxalate and dispergation, the total dissolved Fe at 12 h was slightly increased compared with in samples not dispersed ultrasonically, but the increase was not systematically related to the increase in oxalate/Fe molar ratio (8.8%, 4.1%, 0.29% and 21.3% for oxalate/Fe molar ratios of 0.01, 0.1, 0.2 and to 0.5, respectively). Moreover, for *t* > 12 h, the effect of dispergation was hardly observed. In the experiment at pH 7, similar trends were observed as at pH 4.5, but the level of dissolution was systematically lower than that at pH 4.5. For the sample in the absence of oxalate and not ultrasonically dispersed, dissolved Fe could not be detected over 100 h, while for the dispersed sample, the dissolved Fe was ~0.8 µmmol at 100 h. These observations can be explained by the fact that the ultrasonic dispersion resulted in the increase of small-size ferrihydrite particles, which were then dissolved by proton or oxalate preferentially. Overall, our results suggested that the aggregation state of ferrihydrite hindered proton-promoted dissolution to some extent. Thus, the enhancement of dissolution extent and Fe release rate in this study should be directly related to the addition amount of oxalate.

**Figure 1:**
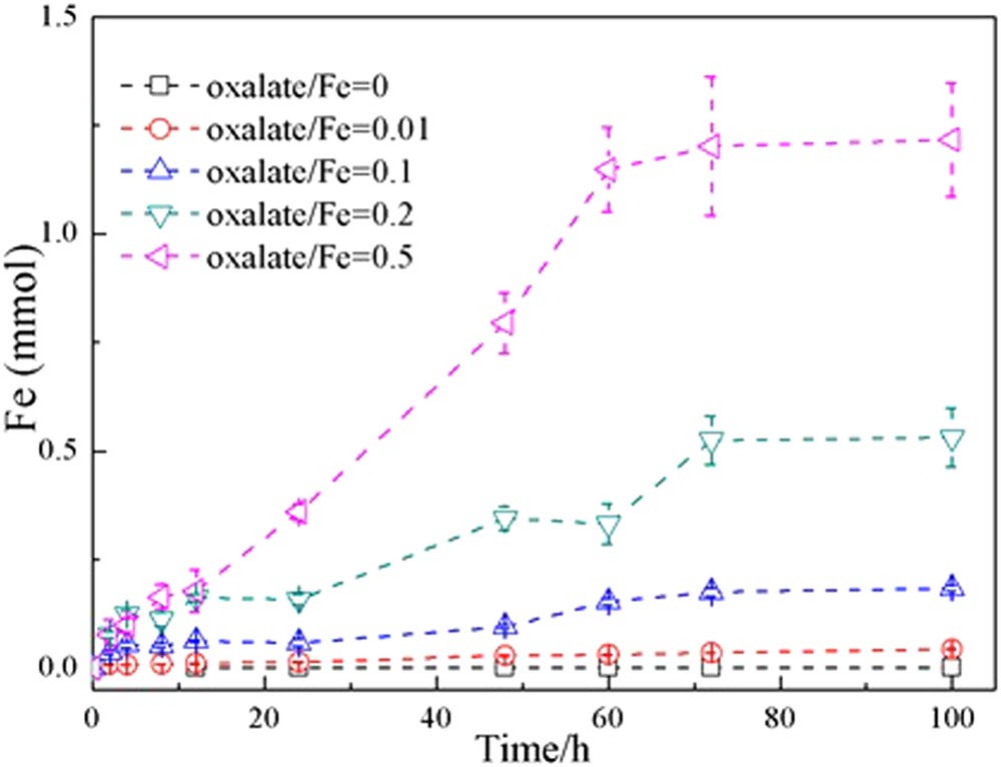
Dissolution kinetics of ferrihydrite for various oxalate/Fe ratios at pH 4.5. The amount of dissolved Fe was obtained from the Fe concentration in the 0.22 μm filtrate and the solution volume.

Figure 1 shows the dissolution kinetics of ferrihydrite at pH 4.5 with the increase of oxalate/Fe ratio from 0 to 0.5. With increasing oxalate/Fe molar ratio (0.01, 0.1, 0.2 and 0.5), the dissolution equilibrium was reached at 72 h for all molar ratios and the total dissolved Fe respectively accounted for 1.1%, 5.8%, 17.5% and 40.0% of the initial total Fe amount (3 mmol). The dissolution rate significantly increased (from 0.04 to 17.2 μmol/h) with increasing oxalate/Fe ratio.

**Figure 2:**
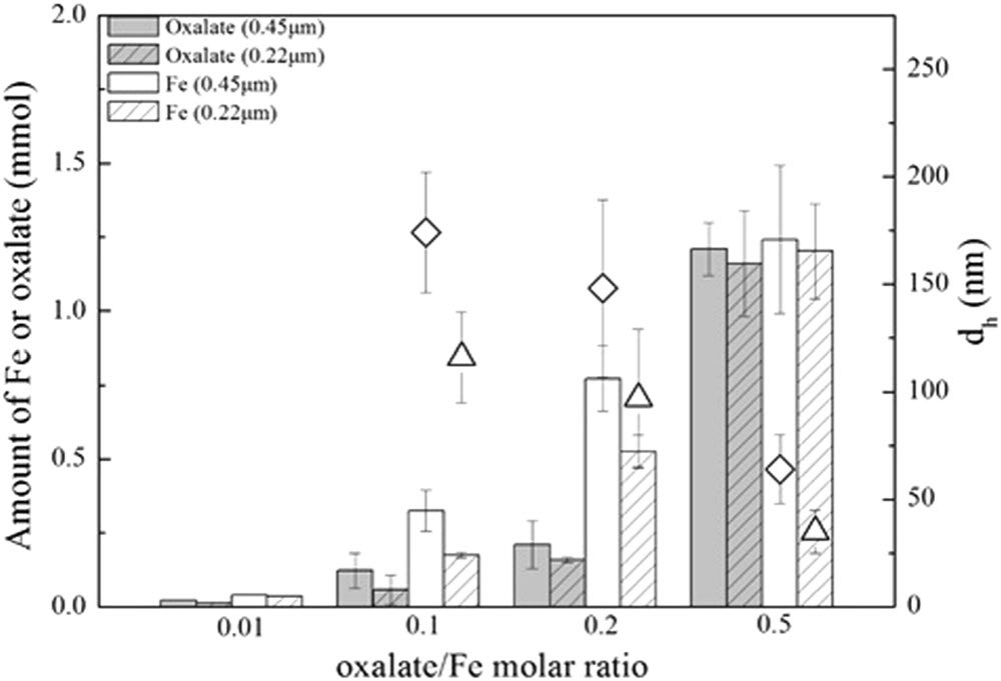
Amounts of Fe and oxalate in 72-h sample filtrates. Diamonds and triangles indicate hydrodynamic diameters (d_H_) of ferrihydrite particles in the 0.45 and 0.22 μm filtrates. Error bars depict standard deviations of three measurements of three replicate experiments.

The 72-h samples were individually passed through filters with pore sizes of 0.45 and 0.22 μm, and their dissolution characteristics were further investigated. As shown in Fig. 2, for the samples with oxalate/Fe ratios of 0.1 and 0.2, the iron amount in 0.45 um filtrate was higher than that in 0.22 um filtrate; and for the samples with the oxalate/Fe ratio of 0.5, the Fe amounts in 0.45 and 0.22 μm filtrates were similar. With increasing oxalate/Fe molar ratio, the average hydrodynamic diameters of ferrihydrite particles decreased from 174 ± 28 to 64 ± 16 nm in 0.45 μm filtrates, and from 116 ± 21 to 35 ± 10 nm in 0.22 μm filtrates. In addition, for samples with oxalate/Fe ratios of 0.1 and 0.2, the Fe amount in the filtrates was at least triple of the oxalate amount. According to the Fe(III)-oxalate speciation at pH 4.5 in Table S2, [Fe(C_2_O_4_) _3_]^3−^ or [Fe(C_2_O_4_) _2_]^−^ was the dominant species at oxalate/Fe ratios of 0.1 and 0.2. Therefore, the oxalate/Fe molar ratio in the filtrates was expected to be about 3 or 2 if dissolution was the only disintegrating process of ferrihydrite aggregates. However, the oxalate/Fe molar ratio was measured to be about 0.3 in the filtrates, implying the stable presence of oxalate-coating ferrihydrite colloidal particles in the filtrates.

The measured oxalate/Fe molar ratio in the filtrates (Fig. 2) first decreased (initial molar ratio 0.01–0.2) and then increased (initial molar ratio 0.2–0.5), which can be explained by ferrihydrite polydispersity. Ferrihydrite consists of a small amount of small-size particles and a large amount of aggregates^4^. At the initial oxalate/Fe molar ratios of 0.01–0.2, oxalate molecules were preferentially adsorbed onto the surfaces of the initially present small-size particles, which considerably affected the dissolution of these ferrihydrite particles^4,38^. With the initial oxalate/Fe ratio increasing from 0.1 to 0.2, both the disintegration and dissolution of small-size particles were substantially enhanced (see Fig. 1), resulting in the release of more particles with smaller size into the filtrates. Thereby, oxalate/Fe molar ratios in the filtrates decreased from 0.01 to 0.2. At the oxalate/Fe ratio of 0.5, excess oxalate could be adsorbed onto the aggregates, and resulted in the dissolution of the aggregates into Fe(III) bound to oxalate, which passed through the filter and entered into the filtrate, and resulted in an increase of the oxalate/Fe ratio from 0.2 to 0.5.

**Figure 3:**
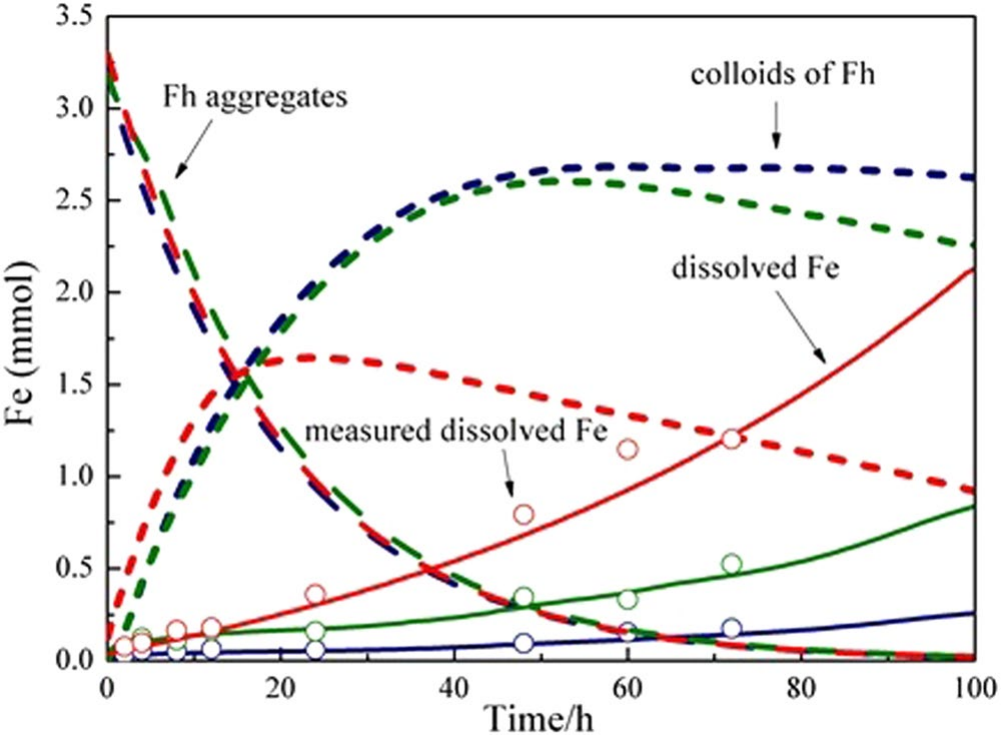
Modeling results of ferrihydrite dissolution kinetics with various oxalate/Fe ratios (blue, 0.1; green, 0.2; red, 0.5) under anaerobic and dark conditions respectively. Dash lines, short dot lines, solid lines and empty circle represent lager ferrihydrite aggregates, colloids of ferrihydrite, dissolved Fe and measured dissolved Fe, respectively.

The mathematical modeling of the dissolution results presented in Fig. 1 is shown in Fig. 3. The minimization of the SQR value for the modeling results of dissolved Fe in regard to measurements resulted in a set of dissolution rate constants, among which k_1_, k_2_, and k_3_ were 0.117, 0.001, and 0.002 h^−1^, respectively. As shown in Fig. 3, although various amounts of oxalate were added, the dissolution rate and extent of ferrihydrite were almost the same in the first 12 h, which is in accordance with the model assumption that the addition amount of oxalate mainly has effects on the dissolution of primary ferrihydrite particles with extremely small sizes. Colloidal particles were also generated at same rate regardless of the amount of oxalate. The difference in the initial oxalate amount seemed to mainly affect the further dissolution of colloidal fraction. The oxalate/Fe ratio of 0.1 did not further promote the dissolution of colloids, but at the oxalate/Fe ratio of 0.5, the amount of dissolved Fe increased dramatically at the expense of colloids after 20 h.

### ATR-FTIR spectroscopy of aqueous oxalate and Fe(III)-oxalate complexes

**Figure 4:**
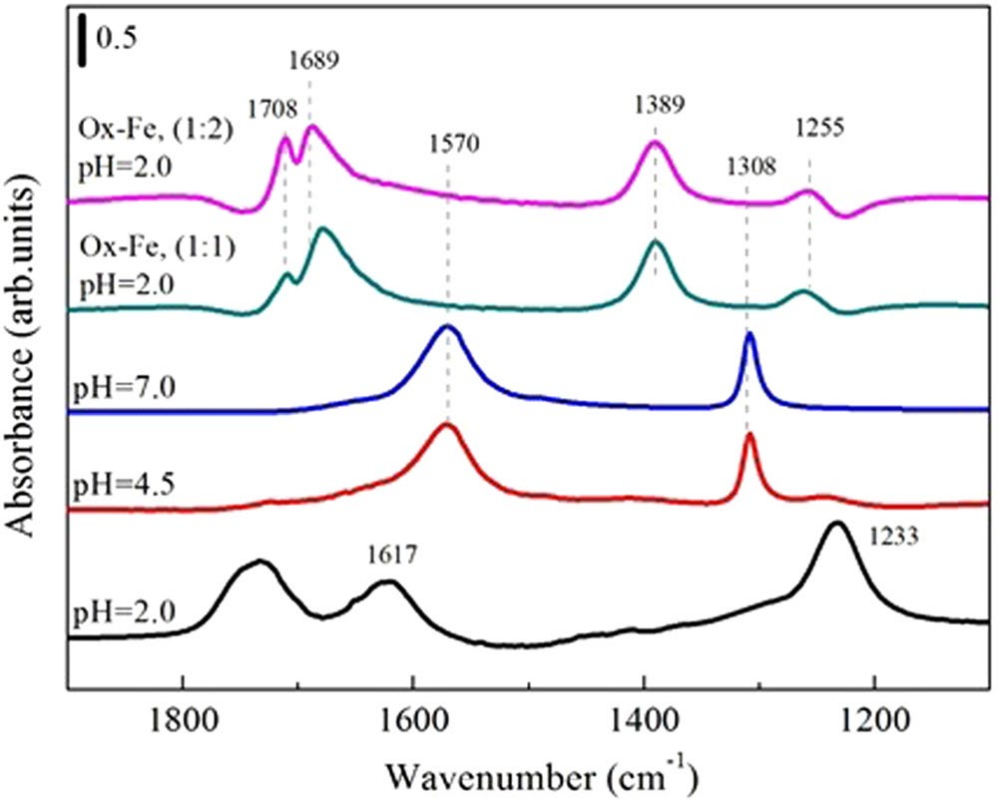
ATR-FTIR spectra of an aqueous oxalate at different pH, and oxalate mixed with Fe(NO_3_)_3_ at the ratios of 1:1 and 1:2 under pH 2.0. The assignments of the spectral features are listed in Table 1.

As a reference, the absorption spectra of aqueous oxalate and Fe(III)-chelated oxalate were shown in Fig. 4. The assignments of the peaks to each chemical species were listed in Table 1, and the speciation of aqueous oxalate-Fe(III) complexes with oxalate/Fe of 1:1 or 1:2 was shown in Table S2. Briefly, oxalic acid HOOC-COOH (H_2_C_2_O_4_) has carboxylic acid functional groups with pK_a1_ = 0.97 and pK_a2_ = 3.57^51^. With a change in the dominant chemical species at specific pH, the peak intensities and positions would be changed. The deprotonated species C_2_O_4_^2−^ was the dominant species at neutral pH. The ATR-FTIR spectra for C_2_O_4_^2−^ showed two strong peaks at 1570 and 1308 cm^−1^ arising from symmetric [ʋ_s(C-O)_] and asymmetric [ʋ_a(C-O)_] stretching modes respectively, which were also retained at pH 4.5. With the decrease of pH value to 2.0, singly-deprotonated species HC_2_O_4_^−^ made the absorption peaks at 1570 and 1308 cm^−1^ shift to 1617 and 1233 cm^−1^, respectively. Meanwhile, the protonated species H_2_C_2_O_4_ displayed stretch absorptions at 1733 cm^−1^ stemming from carbonyl group stretching vibration.

**Table 1:** Assignment of vibrational modes of chemical species of oxalate from ATR-FTIR spectra shown in Figs 4 and 5.

	[ʋ_s(C=O)_]	[ʋ_aC=O)_]	[ʋ_s(C-O)_] + [ʋ_(C-C)_]	[ʋ_aC-O)_]	ref.
**Experimental observations**					
C_2_O_4_^2−^, pH 7.0	—	—	1570	1308	this study
C_2_O_4_^2−^, pH 4.5	—	—	1570	1308	this study
C_2_O_4_^2−^, pH 2.0	1733	1617	—	1233	this study
Ox-Fe (1:1), pH 2.0	1708	1677	1389	1260	this study
Ox-Fe (1:2), pH 2.0	1708	1689	1389	1255	this study
Oxalate + ferrihydrite	1708	1680	1412	1276	this study
**Theoretical calculation**					
Oxalate + ferrihydrite^MNMD^	1680	1597	1383	1239	^51^
Oxalate + ferrihydrite^BNBD^	1683	1570	1443	1305	^51^
Oxalate + ferrihydrite^MNBD^	1687	1674	1399	1280	^51^

Mononuclear monodentate (MNMD); binuclear bidentate (BNBD); mononuclear bidentate (MNBD).

Compared with the ATR-FTIR spectra of free oxalate ion, the spectra of Fe(III)-chelated oxalate (collected at pH 4.5) presented new spectral features at 1708, 1689, 1389, and 1255 cm^−1^. These spectral features were assigned to [ʋ_(CO2Fe)_], and were in line with those of a standard solution of ferrioxalate with a stable five-membered ring structure (mononuclear bidentate)^52^. The difference in the molar ratio of Fe(III) and oxalate ligand only caused a change of the relative peak intensities in 1680 cm^−1^ region, indicating that Fe(III)-oxalate complexes with different molar ratios have similar structures.

### ATR-FTIR spectroscopy of oxalate adsorbed on ferrihydrite

#### Effect of oxalate/Fe ratio on oxalate complexation

**Figure 5:**
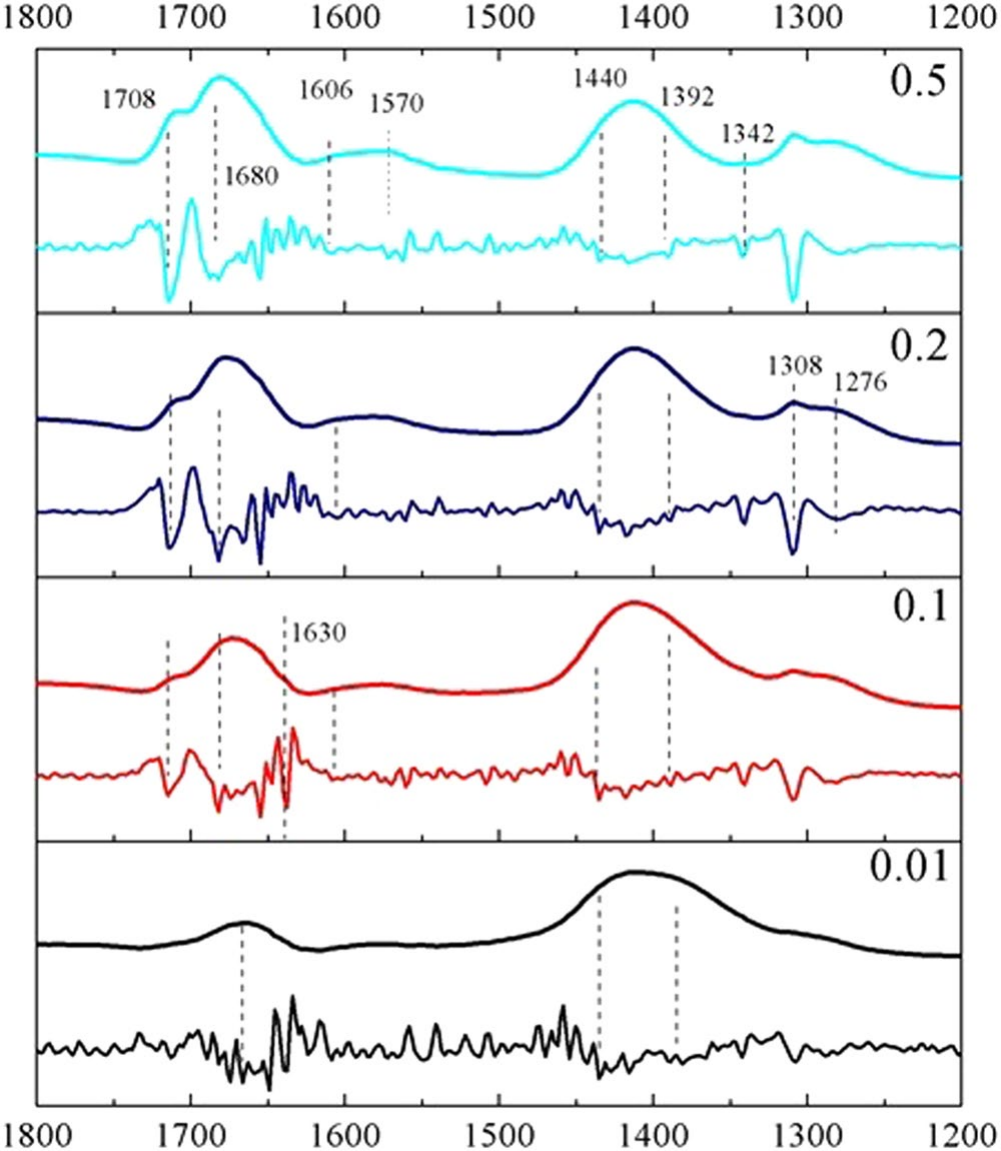
ATR-FTIR absorbance spectra (above curve in each panel) of oxalate adsorbed onto ferrihydrite for the paste samples collected at 72 h and the corresponding second-derivative ATR-FTIR spectra (lower curve in each panel).

Figure 5 shows the ATR-FTIR spectra of wet ferrihydrite pastes at pH 4.5 and different oxalate/Fe ratios. The pastes were collected at a dissolution time of 72 h. Strong peaks were observed at 1680 and 1412 cm^−1^, and weaker ones were found around 1708, 1570, 1308, and 1276 cm^−1^. The relative intensity of the 1708 cm^−1^ peak compared with that at 1680 cm^−1^ increased with increasing oxalate/Fe ratio. Concurrent with this change, the intensity of peaks at 1606 and 1570 cm^−1^ also increased with increasing oxalate/Fe ratio. According to the literature^51^, the peaks at 1708, 1680, 1412, and 1276 cm^−1^ belong to oxalate species adsorbed on ferrihydrite via mononuclear bidentate coordination, and that at 1309 cm^−1^ to H-bonded (or outer-sphere) oxalate. The peak at 1570 cm^−1^ is due to aqueous oxalate species in the diffuse swarm of ions^29^. The bands at 1680 and 1412 cm^−1^ were analyzed in more detail.

Figure 5 also shows the second-derivative spectra of oxalate adsorbed on ferrihydrite. Throughout the considered range of oxalate/Fe ratios from 0.01 to 0.5, the spectra showed roughly the same features. With increasing oxalate/Fe ratio, the band in the 1680 cm^−1^ region was resolved into three peaks located at 1708, 1675, and 1630 cm^−1^, respectively. The 1630 cm^−1^ peak originated from the deformation mode of water^53^, and asymmetry in its shape suggested strong overlapping of peaks^30^. Additionally, a peak was observed at 1342 cm^−1^ that was not clearly shown in the original spectra, which might result from the carbon dioxide adsorbed on the ferrihydrite^51,53^. At around 1412 cm^−1^, the shape and the position of the second-derivative peaks slightly varied with increasing oxalate/Fe ratio. At least two shoulders were present; the high frequency component at 1440 cm^−1^ predominated at low oxalate/Fe ratio while the high- and low-frequency components (1440 cm^−1^, 1392 cm^−1^) coexisted at high oxalate/Fe ratios. To confirm the presence of different frequencies in the 1412 cm^−1^ region, the original spectrum obtained at oxalate/Fe = 0.5 was subtracted from that at oxalate/Fe = 0.1 (see Figure S4); in the difference spectrum, the residuals at 1440 and 1392 cm^−1^ were clearly observed. Bhandari *et al*.^51^ conducted IR vibration frequency calculations associated with oxalate species adsorbed on a Fe-O cluster. For ease of comparison, these results were also listed in Table 1. The two frequencies at 1440 and 1392 cm^−1^ were very close to the quantum mechanical frequencies of binuclear bidentate and mononuclear bidentate oxalate complexes.

**Figure 6:**
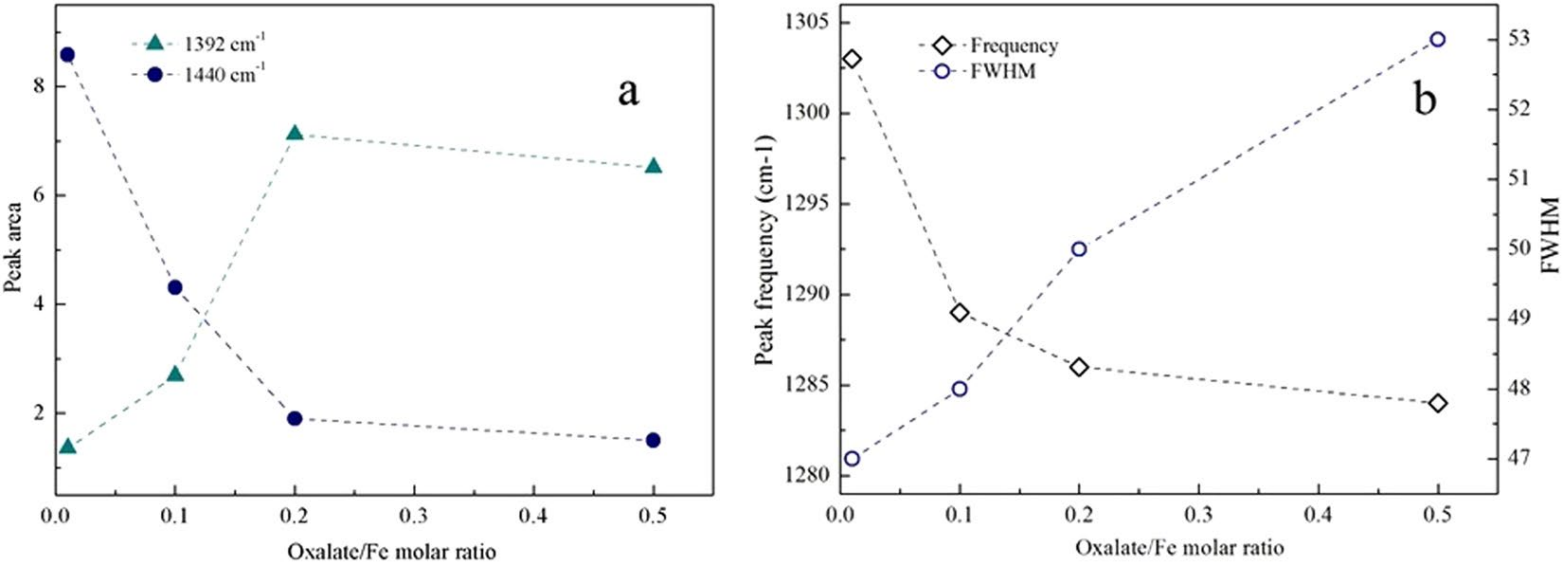
(**a**) Change in peak areas at 1440 and 1392 cm^−1^, (**b**) frequency and full width at half-maximum of the 1280 cm^−1^ peak at different oxalate amounts.

Combining the observations in second-derivative spectra and the theoretical IR vibration frequencies, a Gaussian line shape was employed in the curve-fitting analysis of the overlapping peaks. Such data analysis has been performed before. According to Axe *et al*.^29^, the peak areas of the adsorbed species are proportional to the concentrations of the surface complexes. Fitting of the entire data set resulted in a poor-quality fit. Hence, the entire data set was divided into two parts to be fitted (1750–1500 cm^−1^ and 1500–1200 cm^−1^). The deconvolution results with a high-quality fit (r^2^ = 0.99) are shown in Figure S5. For the present analysis, the 1500–1200 cm^−1^ range was the most interesting. To further investigate the 1440 and 1392 cm^−1^ peaks, the areas of the deconvoluted bands were depicted in Fig. 6a as a function of the ox/Fe ratio. At the oxalate/Fe ratio of 0.01, the area of the 1440 cm^−1^ peak was much larger than that of the 1392 cm^−1^ peak, indicating that the inner-sphere binuclear bidentate oxalate complex was the predominant species. With the oxalate/Fe ratio increasing to 0.2, the area of 1440 cm^−1^ peak decreased and that of the 1392 cm^−1^ peak increased, suggesting that the inner-sphere coordination mode of oxalate gradually shifted from a binuclear bidentate structure to a mononuclear bidentate binding geometry. For the oxalate/Fe ratio of 0.5, the band area was similar to that at oxalate/Fe ratio of 0.2 and inner-sphere mononuclear bidentate oxalate complexes were the predominant species.

In addition, both the frequency and full width at half maximum (FWHM) of the 1280 cm^−1^ peak ([ν_a_(C-O)]) also exhibited some changes with increasing oxalate/Fe ratio. In Fig. 6b, both the frequency and the FWHM are plotted as a function of the oxalate/Fe ratio. At oxalate/Fe ratio of 0.01, the peak frequency was the highest (1303 cm^−1^) and the FWHM was the lowest. The frequency was consistent with the quantum mechanical frequency of binuclear bidentate oxalate complexes^51^ and the narrow FWHM indicated a low heterogeneity of the binding. With increasing oxalate/Fe ratio, the frequency gradually shifted to 1285 cm^−1^ (mononuclear bidentate) and the FWHM increased gradually. The increase in the FWHM could be attributed to an increase in the overall heterogeneity of the occupied surface binding sites with increasing ox/Fe ratio.

In summary, at least five different species were present at or near the ferrihydrite surface: (1) adsorbed carbon dioxide (1342 cm^−1^) at all oxalate/Fe ratios; (2) inner-sphere species with binuclear bidentate binding geometry (1440 cm^−1^) at oxalate/Fe ratios of 0.01–0.1; (3) inner-sphere species with mononuclear bidentate binding geometry (1392 cm^−1^) at oxalate/Fe ratios of 0.2–0.5; (4) outer-sphere species (1309 cm^−1^) at the oxalate/Fe ratios of 0.1–0.5; (5) aqueous oxalate species in the diffuse swarm of ions (1570 cm^−1^).

#### Effects of dissolution time on ATR-FTIR spectroscopy

**Figure 7:**
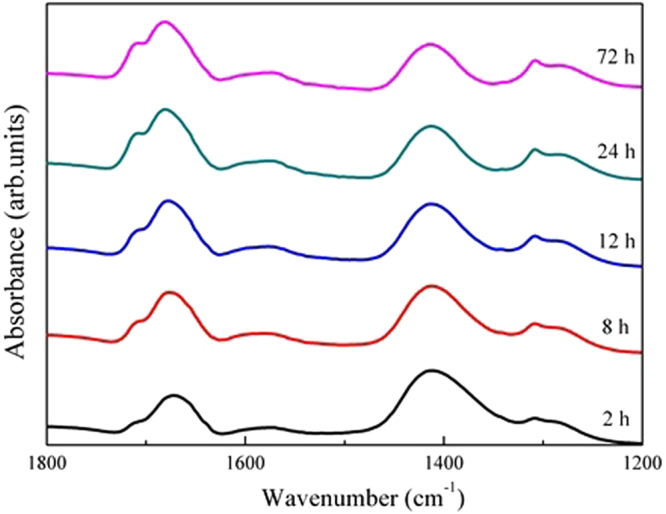
ATR-FTIR spectra of oxalate adsorbed onto ferrihydrite with time for the paste samples with the oxalate/Fe ratio of 0.5.

**Figure 8:**
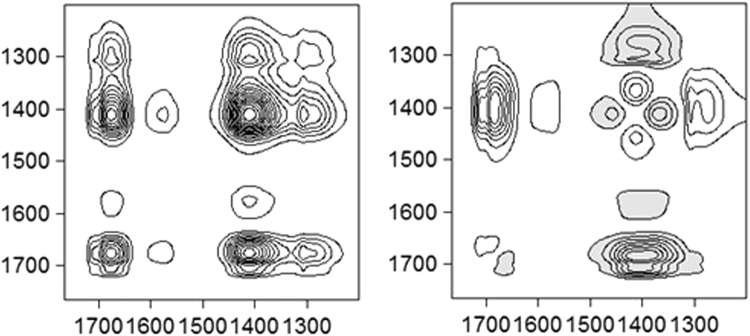
Synchronous contour plots (left) and asynchronous contour plots (right) obtained from the time-dependent ATR-FTIR spectra of the paste samples with the oxalate/Fe ratio of 0.5. The shaded and unshaded areas in the 2D spectra represent negative and positive peaks, respectively.

Figure 7 shows the time-dependent ATR-FTIR spectra of the wet pastes collected from 2 h to 72 h at the oxalate/Fe ratio of 0.5. With increasing reaction time, the absorption intensities were increased to different extents. However, the spectroscopic observation was not sensitive sufficiently to reveal the dynamic changes of different oxalate species with time. To clarify the transformation sequence of different oxalate species adsorbed on ferrihydrite, 2D-FTIR correlation spectroscopy analysis was performed and the results are displayed in Fig. 8.

The synchronous contour plot displays three diagonal peaks at 1309, 1412, and 1685 cm^−1^, which represent the overall susceptibility of the corresponding spectral region to the change in spectral intensity with increasing reaction time. In addition, a small and not obvious auto-peak at 1575 cm^−1^ was observed. The cross peaks at (1675, 1575/1412/1309), (1575, 1412), and (1412, 1309) all exhibited positive signs in the synchronous map, suggesting that these peaks originated from the same responses of spectral intensities to time perturbation.

Asynchronous map was employed to investigate the sequential order of specific events along external perturbations. Cross peaks at (1675, 1412/1309), (1575, 1412), and (1412, 1309) indicated asynchronous behaviors of the intensity variations. No cross peak was observed at (1675, 1575), indicating that these two bands changed simultaneously. According to Noda’s rule, the sequence of band variation with time follows the order of 1675 → 1309 → 1412 cm^−1^. Asynchronous behaviors of peaks at 1675 and 1412 cm^−1^ might be related to the dissolution of ferrihydrite aggregates (see discussion below).

## Discussion

### Oxalate speciation at ferrihydrite/water interfaces

According to Michel *et al*.^54^, the ideal structure of ferrihydrite consists of three types of Fe sites (60% Fe1, 20% Fe2, and 20% Fe3). Fe1 and Fe2 are octahedrally coordinated, while Fe3 is tetrahedrally coordinated. Recently, Hiemstra proposed a modified Michel model (surface depletion model)^55^, in which the ferrihydrite consists of a defect-free mineral core and a defect- and water-rich surface layer. Fe2 and Fe3 polyhedra on the surface are depleted, and Fe1 octahedra are present in rows. These protruding Fe1 octahedra result in two types of surface groups (≡FeOH^0.5−^ and ≡Fe_3_O^0.5−^) with a site density of 5.8 and 1.4/nm^2^, respectively. At low pH, the ≡FeOH^0.5−^ and ≡Fe_3_O^0.5−^ surface groups can be protonated to result in ≡FeOH_2_^0.5+^ and ≡Fe_3_OH^0.5+^ groups, respectively^48^.

Considering the surface structure of ferrihydrite, initially, the inner-sphere adsorbed oxalate is most likely bound to ≡FeOH_2_^0.5+^ sites rather than to ≡Fe_3_OH^0.5+^ sites via a binuclear bidentate mode, which is predominantly due to the relative ease of ligand-substitution for a surface hydroxyl that is bound to only one cation^28^. This binuclear bidentate binding mode of oxalate was also observed with goethite by Filius *et al*.^56^ and with a ferrihydrite film deposited onto ZnSe ATR crystal by Bhandari *et al*.^51^.

Disaggregation of large ferrihydrite aggregates into colloidal particles increases the available surface area and the amount of sorption sites suitable for mononuclear bidentate species, as confirmed by the fact that the area under the 1392 cm^−1^ peak gradually became dominant with increasing oxalate/Fe ratio (Fig. 6a). Dissolution of the defective surface layer following the formation of mononuclear bidentate species would result in the exposure of defect-free mineral core and more ≡Fe_3_OH^0.5+^ sites, causing a gradual transition of the coordination from aqueous oxalate to outer-sphere and then to inner-sphere adsorption mode. As indicated in Fig. 5, excess oxalate was present dominantly as aqueous oxalate species in the diffuse swarm of ions (i.e., at the outer Helmholtz plane and beyond). With increasing oxalate/Fe ratio or increasing reaction time, the intensities of the peaks at 1607 and 1570 cm^−1^ also increased. Similar observations have been reported in previous studies of oxalate adsorption on boehmite^28,29^. Axe and Persson^29^ ascribed these changes of peak shape and position to the asymmetric solvation effect on the oxalate adsorbed in an outer-sphere mode. Here, we also ascribed the increase of the peak at 1607 cm^−1^ to the formation of an outer-sphere complex. At a low oxalate/Fe ratio, outer-sphere oxalate adsorption would be less favored because the ferrihydrite surface charge becomes more negative due to inner-sphere oxalate adsorption. With the progress of dissolution reaction, outer-sphere adsorption may occur through hydrogen bonding interactions involving surface ≡Fe_3_OH^0.5+^ groups. A similar conclusion was made for the adsorption of oxalate on goethite micro- and nano-rods at pH 3 by Cwiertny *et al*.^20^, who showed that more outer-sphere complexes were adsorbed on smaller size particles. Therefore, 2D FTIR correlation spectroscopy demonstrated the sequence of oxalate speciation events at the ferrihydrite/oxalate solution interface with reaction time.

### Impact of oxalate adsorption on dissolution behavior of ferrihydrite

Inorganic ligands adsorbed in an binuclear bidentate mode tend to inhibit mineral dissolution, whereas ligands adsorbed as mononuclear bidentate complexes can enhance dissolution^57^. Our ATR-FTIR results showed that the aggregation state of ferrihydrite determines the surface coordination mode of oxalate, which in turn affects the dissolution behavior of ferrihydrite.

At the oxalate/Fe ratio of 0.01, no ferrihydrite disaggregation was detected (Fig. 2). This was ascribed to the fact that after the adsorption of small amounts of oxalate to ferrihydrite aggregate surfaces, the positive surface charge of pure ferrihydrite is neutralized, resulting in the domination of attractive forces over repulsive forces^58^. At oxalate/Fe ratios of 0.1–0.2, the hydrodynamic diameters of ferrihydrite particles and mathematical simulation results showed that oxalate coating resulted in the disaggregation of large ferrihydrite aggregates into smaller colloidal particles with hydrodynamic diameters of 97–147 nm (Figs 2, 3). A recent study has shown that citrate coating on ferrihydrite may cause electrostatic repulsion between small sub-aggregates within large ferrihydrite aggregates and the formation of a stable colloidal suspension^38^. In our experiments, the electrostatic repulsion probably stemmed from binuclear bidentate oxalate complexes. At the oxalate/Fe ratio of 0.5, the hydrodynamic diameters of ferrihydrite particles further decreased (35–64 nm) and the amount of dissolved Fe increased markedly. ATR-FTIR results showed that the inner-sphere mononuclear bidentate oxalate complexes on ferrihydrite surface dominated over inner-sphere binuclear bidentate complexes (Fig. 6a). Here, the enhancement of colloidal particles dissolution was ascribed to the formation of the mononuclear bidentate oxalate complexes. Furrer and Stumm^21,22^ have proposed that mononuclear bidentate oxalate complexes resulted in the polarization and weakening of bridging metal-oxygen bonds in positions trans to each metal-anion bond, which makes the release of complexed metal cations from the surface relatively easy. The mononuclear bidentate oxalate complexes also caused the transfer of considerable electron density into the coordination sphere of the surface metal cations^21^. In this scenario, reductive dissolution could occur, and Fe(II) as the reduction product of Fe(III) at the ferrihydrite surface is more susceptible to be released into the solution.

**Figure 9:**
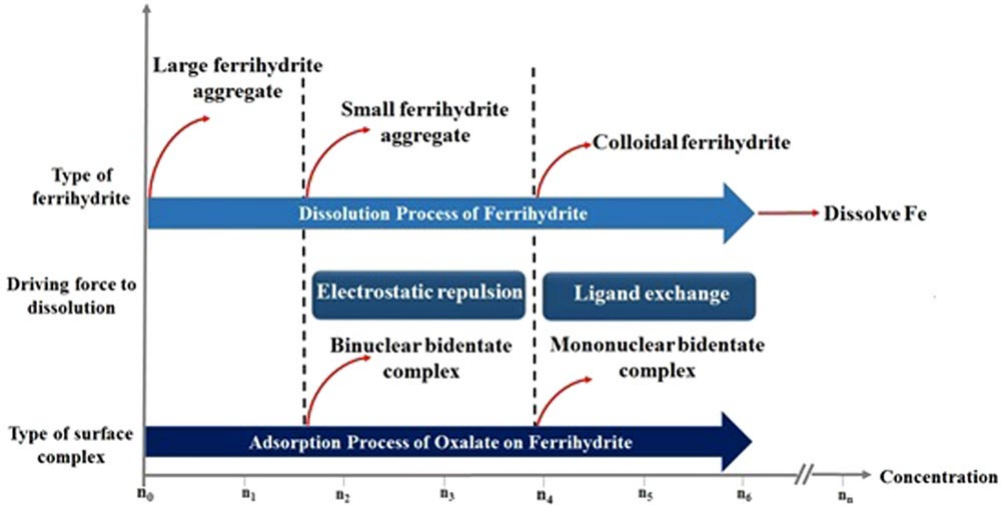
Schematic illustration of the relationship between oxalate coordination modes and the dissolution behavior of ferrihydrite.

Based on these results, the proposed relationship between oxalate coordination modes and the dissolution behavior of ferrihydrite is shown in Fig. 9. At sufficiently low oxalate concentrations, the oxalate is strongly bound to the surface of ferrihydrite aggregates in the form of binuclear bidentate complex, which causes electrostatic repulsion, resulting in the liberation of colloidal particles from the large aggregates. With the increase in the available surface area, the oxalate is bound to the surface of ferrihydrite in the form of mononuclear bidentate complex, which leads to the further breaking-up of large aggregates and dissolution of colloidal particles into dissolved Fe.

## Environmental Implications

In this study, we concluded that the change in oxalate concentrations or dissolution time is likely to induce the change in the surface coordination mode of oxalate, which results in the change in the ratio of dissolved and colloidal fractions of Fe during the dissolution of ferrihydrite aggregates. A chemical perturbation caused by the change in oxalate concentrations has been observed in certain environments. For instance, in soils where Chinese fir of different developmental stages are planted, the oxalate content is substantially higher in mature forest soil than in young forest soil and decreases with increasing soil depth in soils form forests of the same age^59^. For another example, the oxalate content in the agricultural soil of Chinese loess plateau increases substantially (from 150 to 900 µg/kg) during the decay period of corn^60^. A chemical perturbation could cause different environmental behaviors of iron hydroxides. First, the disintegration of ferrihydrite aggregates into colloidal particles plays an important role in iron transmission. Second, previous studies have shown that the metal pollutants bound to metal oxides such as Co, Ni and Cu are released in the ligand-promoted and reductive dissolution of metal oxides^4^. The change in oxalate concentration may result in the redistribution of metal pollutants in the solution and available surfaces by controlling the fate of ferrihydrite in the environment.

## Electronic supplementary material

Supplementary Information
