# Supplementary material for: Roles of different types of oxalate surface complexes in dissolution process of ferrihydrite aggregates
Source: Sci Rep. 2018 Feb 1;8:2060. doi: 10.1038/s41598-018-20401-5 (PMC5794740; doi:10.1038/s41598-018-20401-5)
Supplement: Supplementary file 1 — Supplementary Information [file 41598_2018_20401_MOESM1_ESM.doc]

**Supporting Information**

**Roles of different types of oxalate surface complexes in dissolution process of ferrihydrite aggregates**

Fengyi Li^†^, Luuk Koopal^‡^, and Wenfeng Tan^†*^

^†^*Key Laboratory of Arable Land Conservation (Middle and Lower Reaches of Yangtze River), Ministry of Agriculture, College of Resources and Environment, Huazhong Agricultural University, Wuhan 430070, People’s Republic of China*

^‡^*Physical Chemistry and Soft Matter, Wageningen University and Research, Stippeneng 4 (Helix), 6708 WE, Wageningen, The Netherlands.*

*Corresponding author: Wenfeng Tan (E-mail: [wenfeng.tan@hotmail.com](mailto:wenfeng.tan@hotmail.com))


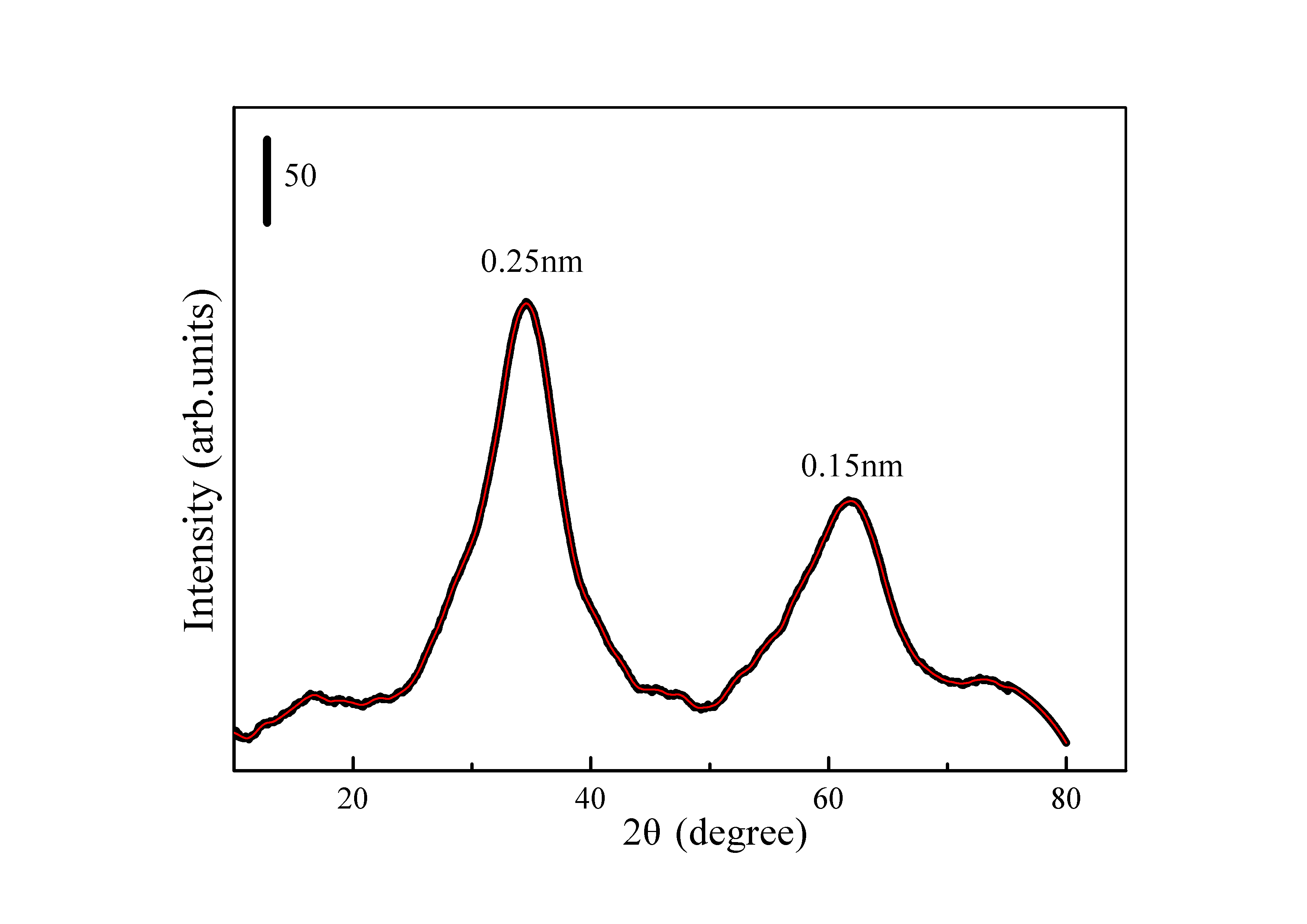


**Figure S1.** X-ray diffractograms of ferrihydrite and the Rietveld fitting results. The black line represents the observed data, and the red line is the fitting profile.

Table S1. Cell parameters obtained by Rietveld fitting for ferrihydrite.

| a | c | crystallite size | crystallite volume | R_wp_ |
| --- | --- | --- | --- | --- |
| (Å) | (Å) | (nm) | (Å^3^) | (%) |
| 5.653 | 9.178 | 2.6 | 254 | 1.67 |


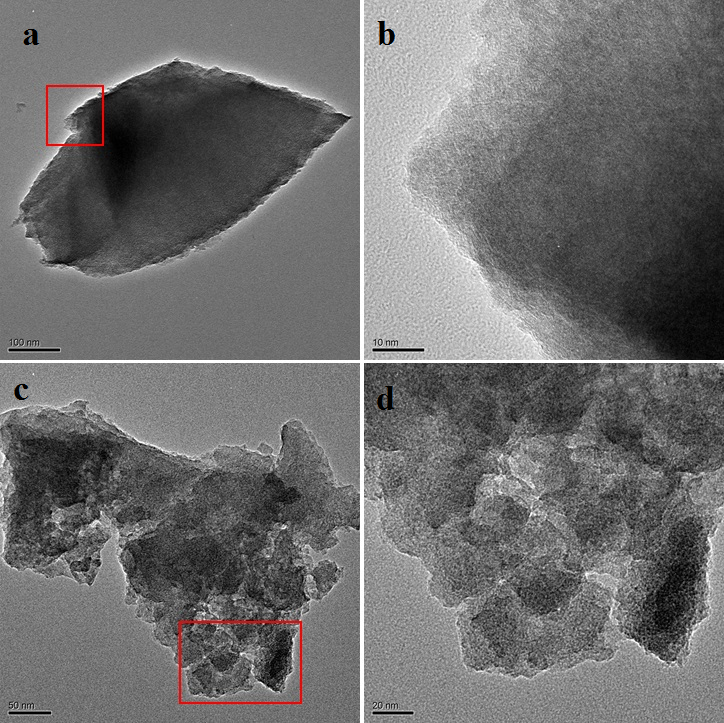


**Figure S2** TEM images of the ferrihydrite before and after dissolution. (a) and (b) TEM images before dissolution; (c) and (d) TEM images after the addition of oxalate (oxalate/Fe ratio of 0.5) collected at 48 h.


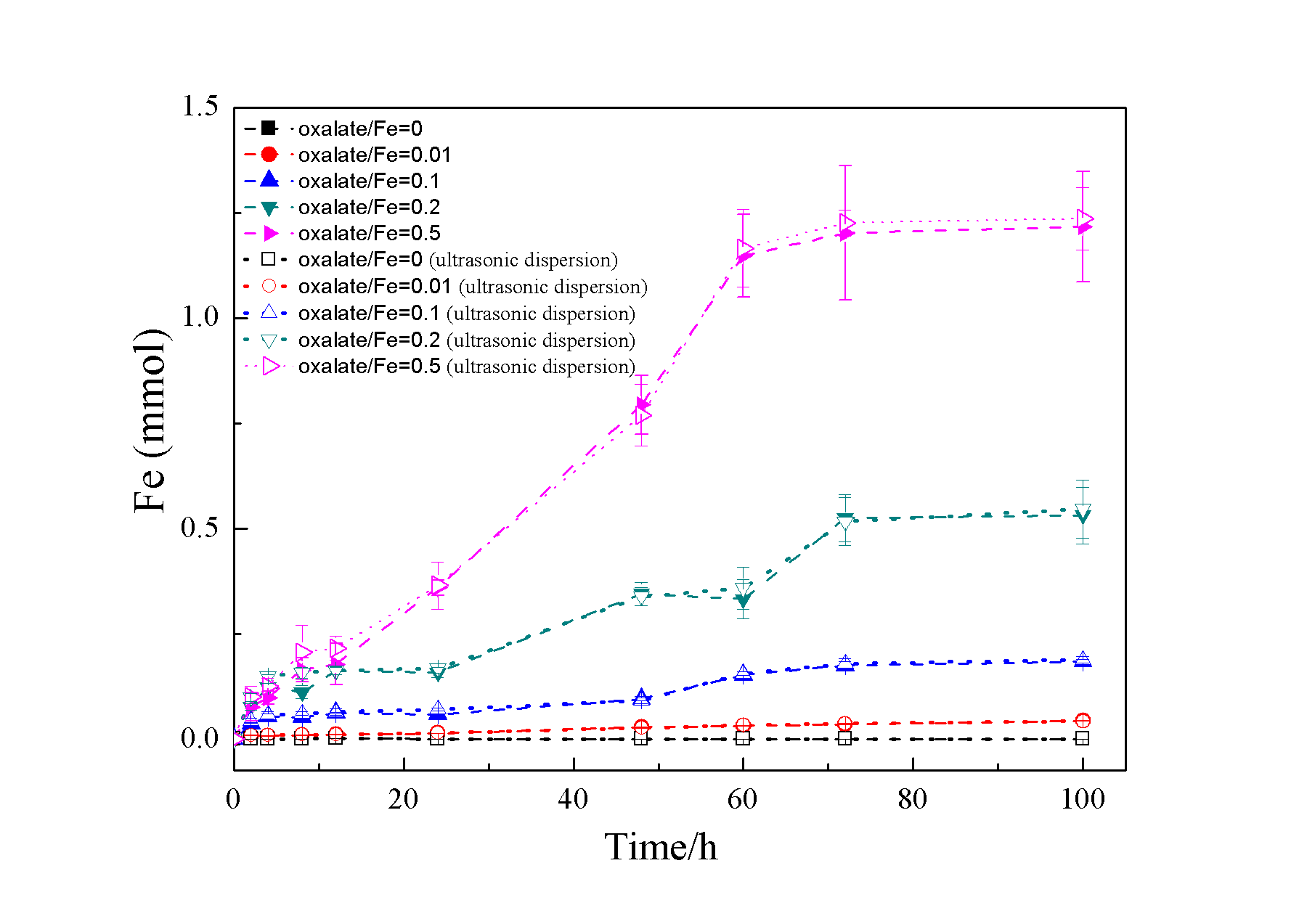


pH 4.5


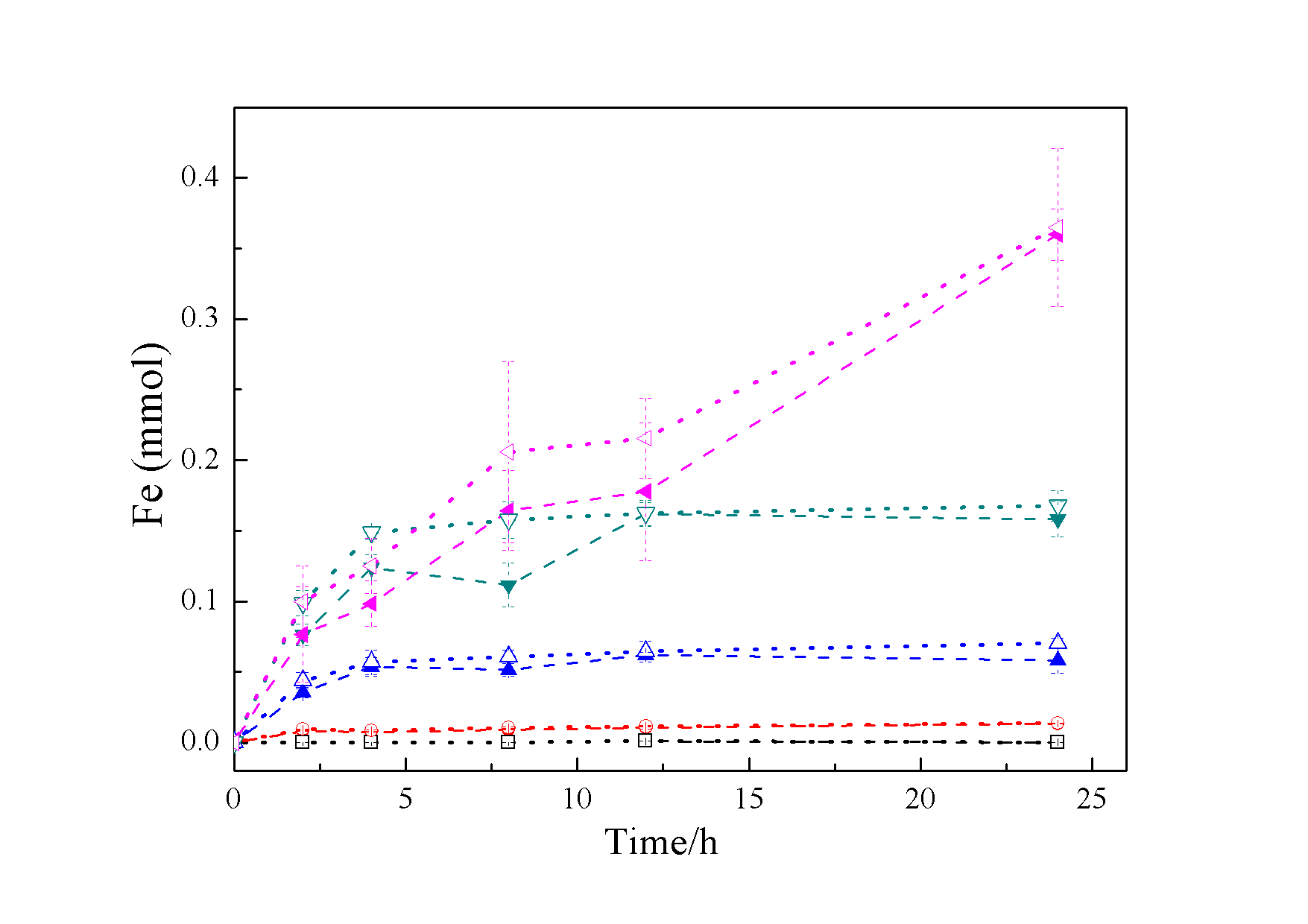


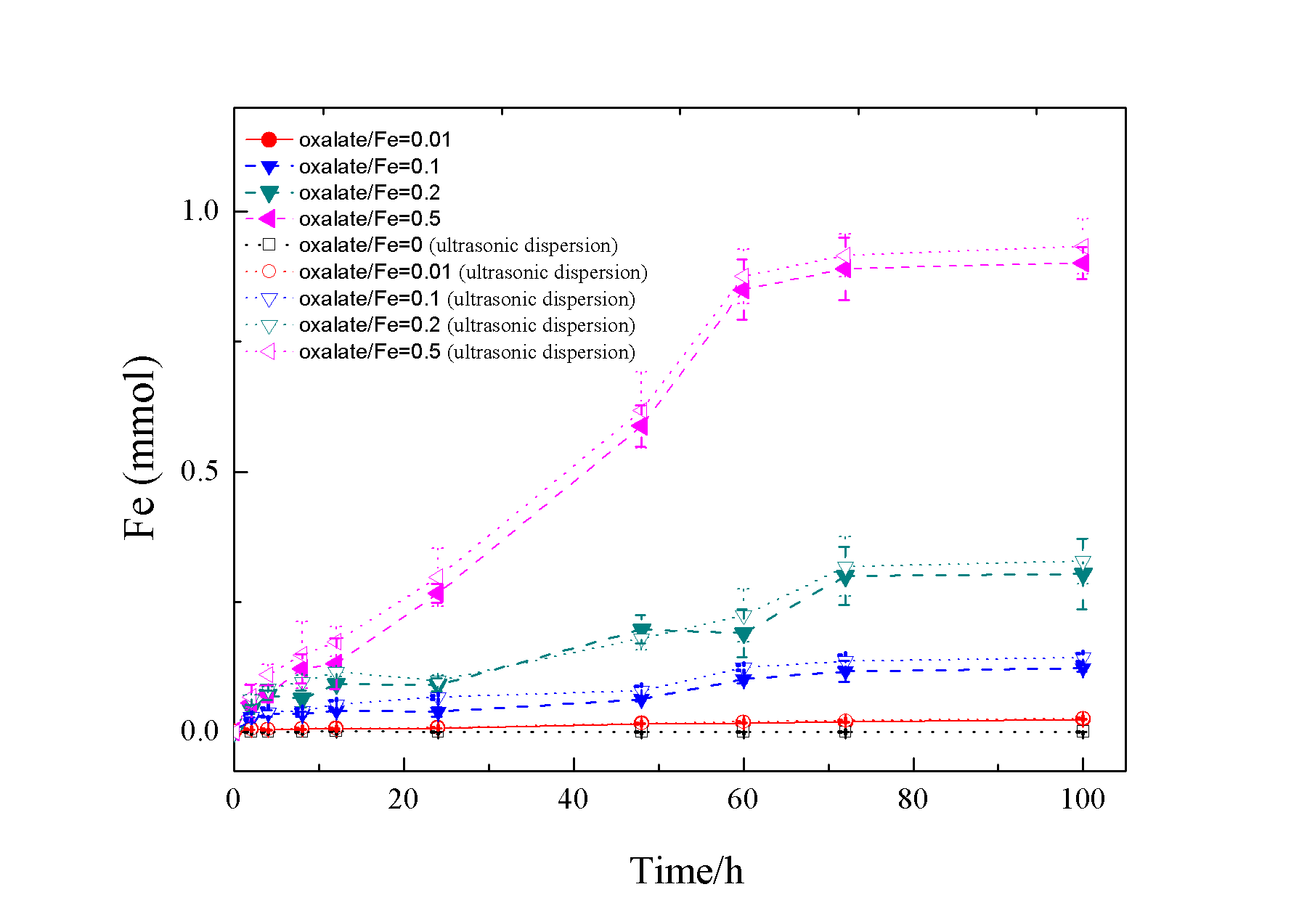

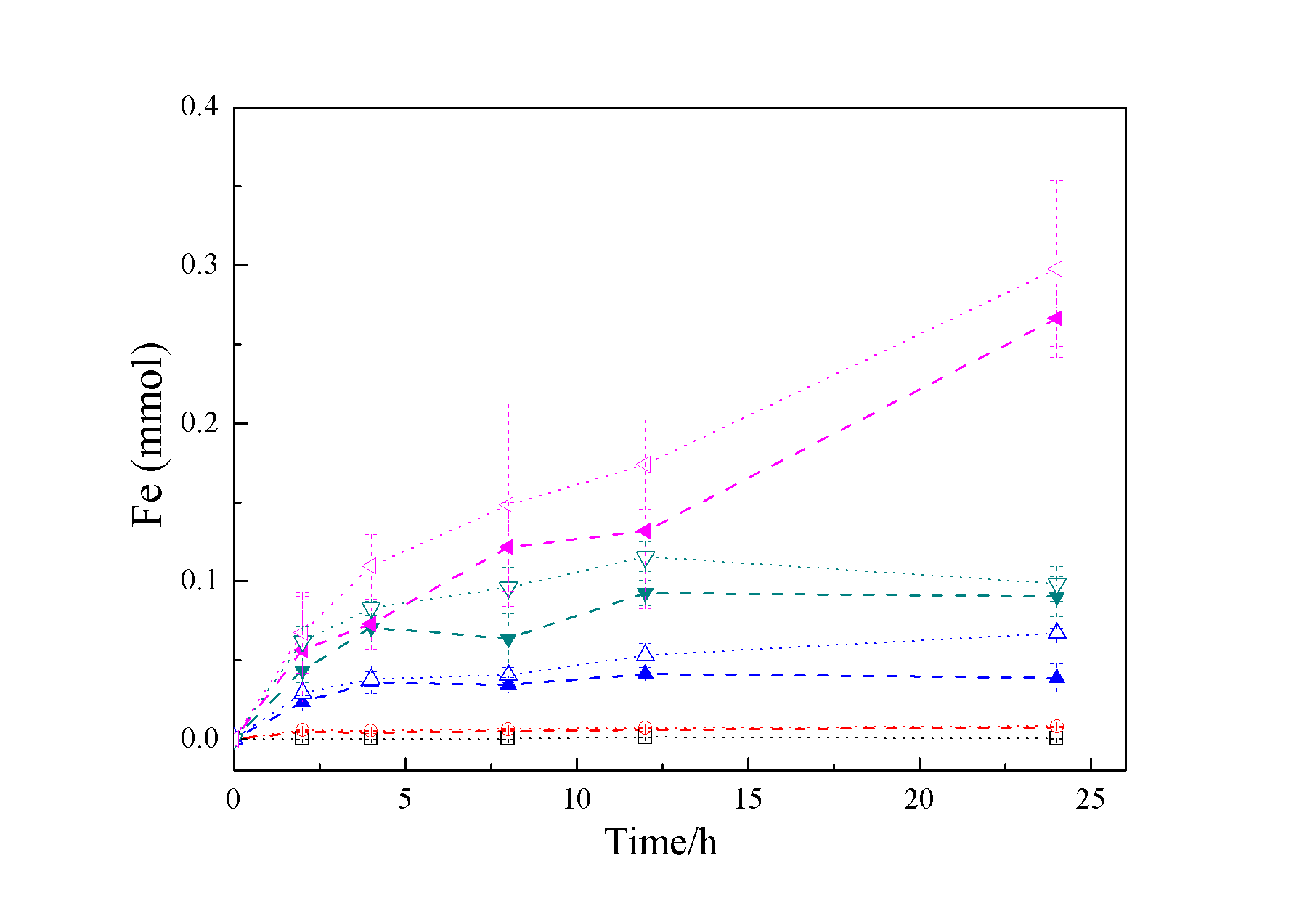


pH 7.0

**Figure S3** Dissolution kinetics of ferrihydrite with various oxalate/Fe ratios at pH 4.5 and pH 7.0. Dot lines represent dissolution kinetic curves for the sample which was dispersed for 30 min by an ultrasonic method prior to the dissolution; Dash lines represent dissolution kinetic curves for the sample which was not dispersed.


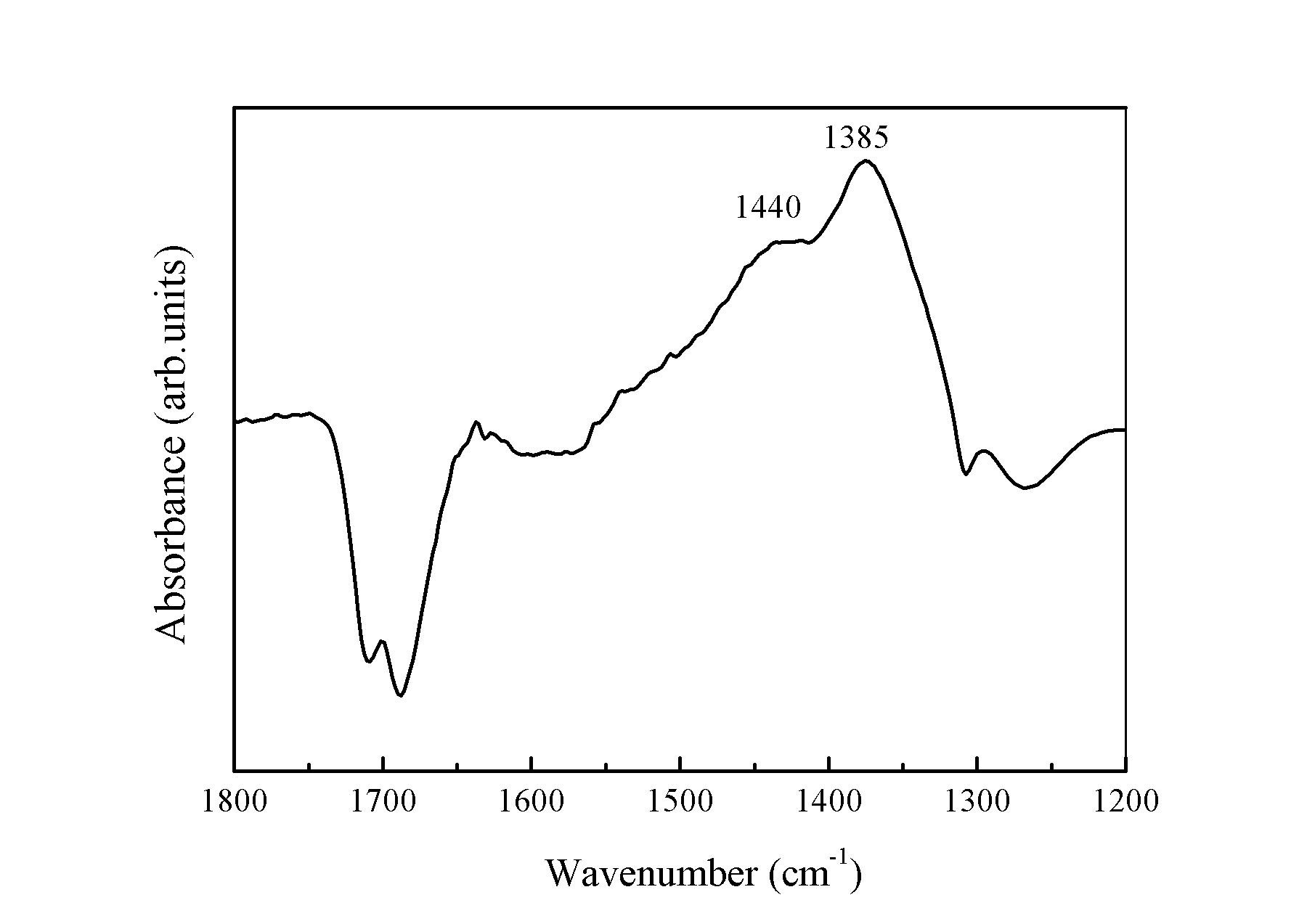


**Figure S4** Difference spectrum obtained by subtracting the original spectrum of the sample with oxalate/ Fe ratio of 0.5 from that with oxalate/ Fe ratio of 0.1.


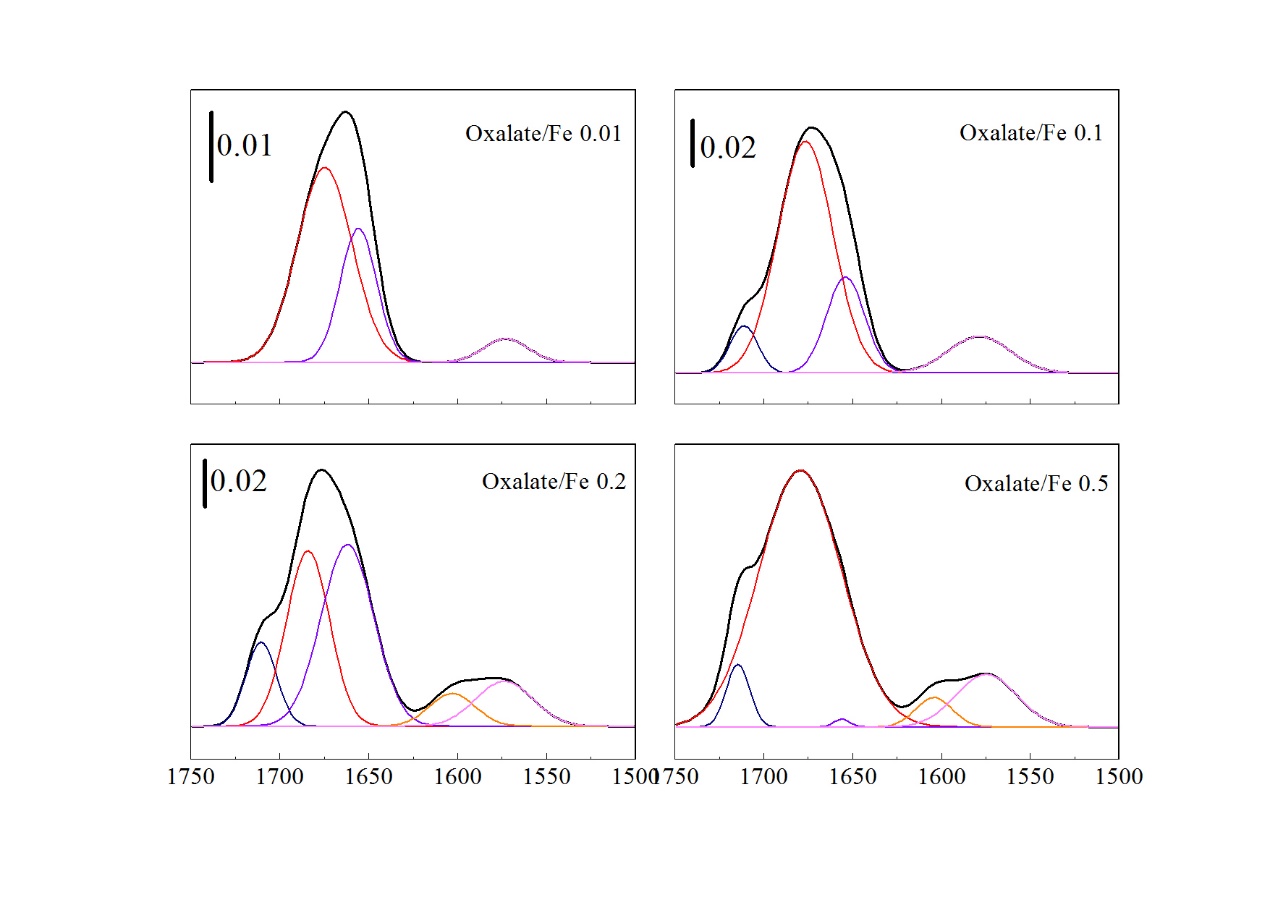


(a)


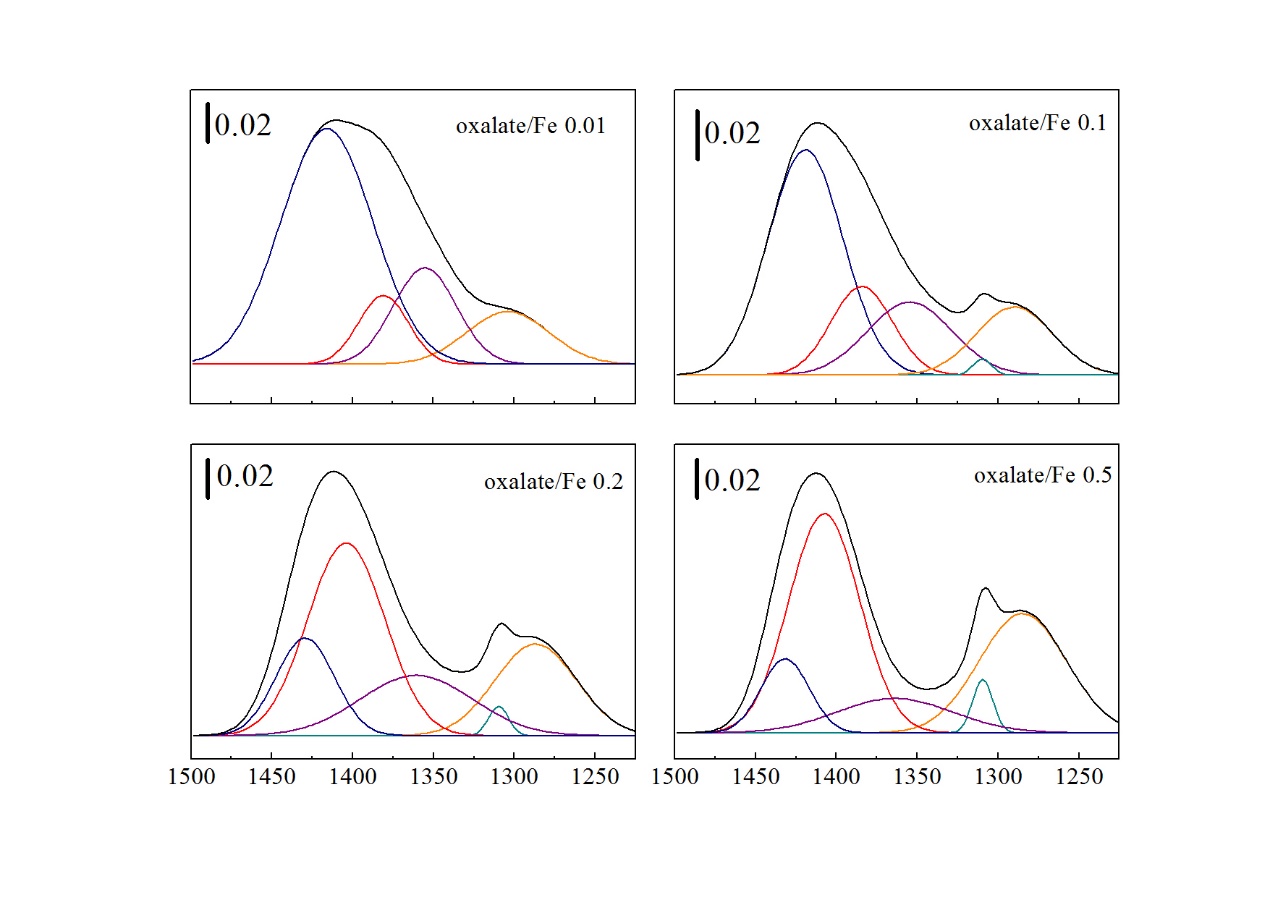


(b)

**Figure S5** Curve-fitting results for spectra from (a) 1700 to 1500 cm^-1^ ;(b) 1500 to 1200 cm^-1^ at various oxalate/Fe ratios.

| Sample | Composition | | | | | | | | | | |
| --- | --- | --- | --- | --- | --- | --- | --- | --- | --- | --- | --- |
|  | Fe | | | | | | Oxalate | | | | |
|  | Fe^3+^ | Fe(OH)^2+^ | Fe(OH)_2_^+^ | Fe-(Oxalate)_3_^3-^ | Fe-(Oxalate)_2_^-^ | Fe-(Oxalate)^+^ | H-Oxalate^-^ | Oxalate^2-^ | Fe-(Oxalate)_3_^3-^ | Fe-(Oxalate)_2_^-^ | Fe-(Oxalate)^+^ |
| oxalate-Fe(1:1, pH=2.0)a | 3.9 |  | 2.5 |  |  | 87.3 | 0.1 |  |  | 12.5 | 87.3 |
| oxalate-Fe(1:2, pH=2.0)b | 31.5 | 16.7 | 0.2 |  |  | 49.5 |  |  |  | 1.0 | 99.0 |
| oxalate-Fe(1:0.53, pH=4.5)c |  | 0.6 | 0.6 | 8.3 | 72.9 | 17.5 | 0.1 |  | 13.3 | 77.3 | 9.3 |
| oxalate-Fe(1:0.37, pH=4.5)d |  |  |  | 65.7 | 33.8 |  | 0.8 |  | 73.0 | 25.0 |  |
| oxalate-Fe(1:0.27, pH=4.5)e |  |  |  | 97.0 | 3.0 |  | 1.8 | 18.0 | 78.6 | 1.6 |  |
| oxalate-Fe(1:0.97, pH=4.5)f | 5.1 | 34.5 | 5.6 |  | 1.7 | 52.3 | 0.1 |  |  | 6.2 | 93.7 |

Table S2 Fe(III)-oxalate speciation

(a)–(b):initial oxalate concentration of 0.5 mmol/L.

(c)–(f): Oxalate/Fe ratio and initial concentrations were set based on measurements in Figure 2.
